# Digital Care for Chronic Musculoskeletal Pain: 10,000 Participant Longitudinal Cohort Study

**DOI:** 10.2196/18250

**Published:** 2020-05-11

**Authors:** Jeannie F Bailey, Vibhu Agarwal, Patricia Zheng, Matthew Smuck, Michael Fredericson, David J Kennedy, Jeffrey Krauss

**Affiliations:** 1 Department of Orthopaedic Surgery University of California, San Francisco San Francisco, CA United States; 2 Hinge Health, Inc San Francisco, CA United States; 3 Division of Physical Medicine & Rehabilitation Stanford University Palo Alto, CA United States; 4 Vanderbilt University Medical Center Nashville, CA United States

**Keywords:** musculoskeletal pain, low back pain, patient engagement, exercise therapy, telemedicine, telerehabilitation, mobile phone

## Abstract

**Background:**

Chronic musculoskeletal pain has a vast global prevalence and economic burden. Conservative therapies are universally recommended but require patient engagement and self-management to be effective.

**Objective:**

This study aimed to evaluate the efficacy of a 12-week digital care program (DCP) in a large population of patients with chronic knee and back pain.

**Methods:**

A longitudinal observational study was conducted using a remote DCP available through a mobile app. Subjects participated in a 12-week multimodal DCP incorporating education, sensor-guided exercise therapy (ET), and behavioral health support with 1-on-1 remote health coaching. The primary outcome was pain measured by the visual analog scale (VAS). Secondary measures included engagement levels, program completion, program satisfaction, condition-specific pain measures, depression, anxiety, and work productivity.

**Results:**

A total of 10,264 adults with either knee (n=3796) or low back (n=6468) pain for at least three months were included in the study. Participants experienced a 68.45% average improvement in VAS pain between baseline intake and 12 weeks. In all, 73.04% (7497/10,264) participants completed the DCP into the final month. In total, 78.60% (5893/7497) of program completers (7144/10,264, 69.60% of all participants) achieved minimally important change in pain. Furthermore, the number of ET sessions and coaching interactions were both positively associated with improvement in pain, suggesting that the amount of engagement influenced outcomes. Secondary outcomes included a 57.9% and 58.3% decrease in depression and anxiety scores, respectively, and 61.5% improvement in work productivity. Finally, 3 distinct clusters of pain response trajectories were identified, which could be predicted with a mean 76% accuracy using baseline measures.

**Conclusions:**

These results support the efficacy and scalability of a DCP for chronic low back and knee pain in a large, diverse, real-world population. Participants demonstrated high completion and engagement rates and a significant positive relationship between engagement and pain reduction was identified, a finding that has not been previously demonstrated in a DCP. Furthermore, the large sample size allowed for the identification of distinct pain response subgroups, which may prove beneficial in predicting recovery and tailoring future interventions. This is the first longitudinal digital health study to analyze pain outcomes in a sample of this magnitude, and it supports the prospect for DCPs to serve the overwhelming number of musculoskeletal pain sufferers worldwide.

## Introduction

### Background

Chronic musculoskeletal pain has vast global prevalence [1] and annual costs in the hundreds of billions of dollars in the United States [2,3]. Musculoskeletal disorders are debilitating and may contribute to the opioid epidemic, as they are the most common noncancer indication for an opioid prescription in the United States [4-6]. Nonsurgical care, including exercise, education, and behavioral health, is universally recommended as the first-line treatment for the majority of chronic musculoskeletal conditions [7] given that it can achieve similar outcomes to surgery with reduced cost and lower risk [8,9]. However, conservative care has significant barriers to effective implementation and requires higher patient engagement to be successful [10,11]. Notably, conservative care administered in a clinical setting is also costly, and ongoing monitoring is often infeasible. Given the growing burden of chronic musculoskeletal pain, a scalable and effective mode of conservative care delivery is needed.

Digital health interventions have the potential to improve conservative care outcomes for chronic musculoskeletal pain by increasing patient engagement through electronic delivery of interventions. This approach can better enable patients to take a proactive role in their treatment and learn to self-manage their chronic pain symptoms. With the ubiquity of smartphones, low-cost sensor technology, and advanced analytical approaches to assess complex health care data, the prospect of digital technology for improved patient care is apparent and is reflected in the growing number of clinical trial protocols and review papers on the topic [12]. Digital therapies are shown to be effective for improving outcomes associated with conditions requiring self-management and behavioral change, such as type 2 diabetes [13], hypertension [14], and insomnia [15]. In addition, patient willingness to seek surgical treatment is shown to decrease following participation in a digital care program (DCP) [16]. Chronic pain, although often difficult to diagnose and treat clinically, is also shown to improve with the aid of digital therapy [10]. For chronic musculoskeletal pain specifically, the DCP in this study was previously evaluated in two randomized control trials and demonstrated effectiveness for improving pain and disability associated with knee pain [17] and low back pain [18]. Although these previous musculoskeletal pain studies show potential for a digital therapeutic approach to improve outcomes, they are limited in sample size (<200 subjects) and real-world effectiveness has yet to be shown. In this study, we assessed engagement and subject-reported outcomes over a 12-week period following enrollment in the DCP in a sample of over 10,000 users with chronic knee or back pain.

### Objectives

This study had two objectives. First, we sought to determine whether the DCP is scalable and effective in a large sample of real-world patients. Given the magnitude of the chronic musculoskeletal pain population, scalability is one of the greatest potential benefits of a DCP, so the efficacy of a DCP in a large sample of real-world patients is important to assess. Key questions include if high levels of engagement can be sustained and if efficacy demonstrated in smaller randomized control trials is maintained in the larger real-world population. On the basis of results from the smaller randomized control trials, we hypothesized that the DCP would improve subject-reported pain over a 12-week period and that engagement with the DCP would be a necessary factor for improvement. A scalable digital intervention for engaging patients with safe conservative therapies for lasting self-management would have the potential to reduce the economic burden and improve the quality of life for a large population of patients.

Second, we sought to analyze the large dataset generated from the DCP to generate novel insights into patient recovery trajectories, which would create an opportunity to develop personalized interventions for individual patients. Little is known about the patient-specific response and rate of improvement for chronic musculoskeletal pain between clinical visits. Patients are typically assessed by clinicians during initial evaluations and, then, at follow-up appointments that may be weeks or months apart. A DCP enables regular (eg, weekly) collection of subject-reported outcomes throughout the recovery process. Statistical modeling methods can then be applied to these large longitudinal datasets to assess the rate of change in outcomes and if baseline data can predict recovery response. In this study, we used statistical modeling on a large longitudinal sample to evaluate nonlinear changes in pain over time and predict subject-specific pain response groups (rapid vs gradual) from baseline demographic data. Understanding how pain improves over time would inform our knowledge of pain recovery, identify variables associated with recovery, and allow for better care of patients unlikely to have rapid pain responses.

## Methods

### Study Design

This was a retrospective cohort study of consecutively recruited participants. Employees and their dependents at 30 participating employers across the United States were invited to complete a web-based application to participate in the Hinge Health DCP. Employees were diverse and included both office and service-based roles such as data analysts, manual laborers, truck drivers, catering staff, and outdoor instructors. Participants with low back or knee pain were recruited through email, direct mail, and posters. The trial was approved by the Western Institutional Review Board and complied with all ethical regulations. Participants provided informed consent and completed the intervention remotely. Each participant participated in 1 of 2 digital care pathways: 1 for chronic knee pain and the other for chronic low back pain. The only differences between the 2 pathways were the specific exercise regimens and some condition-specific education materials (eg, anatomy and surgical options). To mitigate the risks of selection bias, we included all participants who had registered in the Hinge Health program by the cutoff date (May 6, 2019). We were able to verify that the study sample provided adequate power (after correcting for intrauser clustering effects, a sample size of 10,000 gave us a power of 0.97 to detect a 5-point change in our primary outcome with a type 1 error rate of 0.01). A summary of the key attributes of the cohort is provided in Table 1.

Inclusion criteria to qualify for participation in the DCP included being ≥18 years and not >80 years at the time of enrollment, having at least 12 weeks of back or knee pain, and having a baseline visual analog scale (VAS) score for pain greater than 0. Additional inclusion criteria for this study included starting the DCP, defined as completing at least one exercise session or reading 1 educational paper in the first 2 weeks following registration. Participants were excluded during registration by completing a screening questionnaire, which rejected patients with *red flag* symptoms, including signs of fracture, joint instability, infection, cancer, and cauda equina syndrome. Thus, this study included all consecutively qualified participants who enrolled in the DCP between February 6, 2017, and May 6, 2019, meeting the above inclusion and exclusion criteria

**Table 1:** Demographics and outcome measures (N=10,264).

Variables		Baseline				Final		
		Overall	Back pain (n=6468)	Knee pain (n=3796)	Overall		Back pain (n=6468)	Knee pain (n=3796)
Age (years), mean (SD)		43.57 (11.14)	42.58 (10.91)	45.26 (11.33)	N/A^a^		N/A	N/A
BMI, mean (SD)		30.25 (7.42)	29.76 (7.11)	31.09 (7.84)	N/A		N/A	N/A
**Gender**								
	Female, n (%)	5132 (50.00)	4981 (48.53)	5388 (52.49)	N/A		N/A	N/A
**Measures, mean (SD)**								
	Pain (VAS^b^)	45.13 (22.42)	45.81 (22.16)	43.98 (22.81)	14.24 (15.31)		14.23 (15.12)	14.33 (15.59)
	PHQ-9^c^	3.05 (5.34)	3.35 (5.49)	2.54 (5.04)	1.85 (3.97)		2.12 (4.12)	1.43 (3.38)
	PHQ-9^d^	12.01 (4.61)	11.99 (4.56)	12.06 (4.73)	5.05 (5.72)		5.10 (5.73)	4.95 (5.70)
	GAD-7^e^	3.93 (5.50)	4.39 (5.69)	3.15 (5.08)	2.21 (3.83)		2.48 (3.99)	1.77 (3.51)
	GAD-7^f^	11.49 (4.10)	11.56 (4.13)	11.32 (4.04)	4.78 (5.05)		4.84 (5.01)	4.65 (5.12)
	One-year surgery likelihood (0-100)	12.67 (21.55)	9.07 (17.89)	18.80 (25.51)	4.14 (12.44)		2.88 (9.26)	6.26 (16.1)
	WPAI^g^ (0-100)	31.74 (26.79)	34.12 (26.37)	27.54 (27.02)	11.45 (15.60)		12.24 (15.58)	10.17 (15.57)
	KOOS—pain^h^	N/A	N/A	15.23 (6.66)	N/A		N/A	10.04 (5.81)
	Modified von Korff	N/A	15.95 (5.03)	N/A	N/A		7.75 (5.44)	N/A

^a^N/A: not applicable.

^b^VAS: visual analog scale.

^c^PHQ-9: patient health questionnaire 9-item scale.

^d^The mean and SD of the scores in depressed (PHQ-9>5) subjects.

^e^GAD-7: generalized anxiety disorder 7-item scale.

^f^The mean and SD of the scores in anxious (GAD-7>5) subjects.

^g^WPAI: work productivity and activity impairment.

^h^KOOS—pain: knee injury and osteoarthritis outcome score—pain subscale.

### Digital Care Program

Following registration, participants received a tablet computer via mail with the Hinge Health app installed, along with 2 Bluetooth wearable motion sensors with straps and instructions to be placed above and below the painful region during the in-app exercise therapy (ET). In the lower back program, a sensor was placed on the posterior lower back and anterior chest, and for the knee program, a sensor was placed over the anterior tibia and thigh. Sensors utilized standard accelerometer and gyrometer technology (InvenSense MPU-6050, TDK Electronics, Tokyo, Japan) and were used to objectively monitor compliance and performance of exercises. ET sessions comprised light-intensity stretching and strengthening exercises commonly used in clinical practice. The ET sessions were administered using animations and instructional videos to demonstrate how to perform each exercise. While performing the exercise, the app then displayed real-time graphics showing the position of the user’s relevant body parts based on the wearable sensors and indicated if the exercise was within the desired range of movement (see Multimedia Appendices 1 and 2.

Participants were assigned a personal coach and communication was performed via text message, email, or in-app messaging throughout the DCP. Health coaches completed certification through a coaching school approved by the National Board for Health & Wellness Coaching. Coaches attempted to interact with participants via their preferred communication method at least weekly. Phone calls with the coach were also offered to participants up to 3 times during the DCP. Each participant was also placed on a peer support team of 20-30 participants that utilized a discussion forum within the app, as previous qualitative research showed this to be an important feature [19]. All app participation was completed remotely, at times and places chosen by the participant. Each week, participants were instructed to complete at least three sessions of sensor-guided ET, read 2 education papers, and log their symptoms at least twice. Participants were able to complete more ET sessions or read more education papers if desired. Behavior change topics were addressed through education papers and brief interactive modules, and focused on common cognitive behavioral therapy topics, including catastrophizing, active coping methods, and fear avoidance. Additional behavior change mechanisms used in the program included goal setting and tracking. Finally, participants were encouraged to engage in 3 aerobic exercise activities per week and perform up to 4 brief modules based on cognitive behavioral therapy between weeks 3 and 9. Each participant also maintained access to treatment as usual. The app was developed, owned, and sponsored by Hinge Health, Inc.

### Outcomes

The primary outcome was VAS pain for the question “Over the past 24 hours, how bad was your [back/knee] pain?” from 0 (*none*) to 100 (*worst imaginable*). This was asked weekly during the 12-week period immediately after an ET session, and participants also had the option to report VAS unprompted, for a total of up to 2 pain scores per week. Our definition of a minimally important change in VAS pain was a 30% or 20-point decrease from baseline. Secondary outcomes included the patient health questionnaire 9-item scale (PHQ-9, 0-27) for depression, the generalized anxiety disorder 7-item scale (GAD-7, 0-21) for anxiety, the work productivity and activity impairment (WPAI) scale, the knee injury and osteoarthritis outcome score—pain subscale (KOOS—pain, 100-0) for knee pathway participants, the Modified von Korff scale (MvK, 0-100) for back pathway participants [20,21], and surgery likelihood (“What do you think are the chances you’ll have [back/knee] surgery in the next year, in %?”, 0-100%). These secondary outcomes were collected at baseline, 6-weeks, and 12-weeks. Other baseline measurements obtained at week 0 consisted of participants’ age, gender, and BMI. Participants’ engagement with the DCP was measured by recording the number of ET sessions completed, the number of coaching interactions, and the number of education papers read. Each coaching interaction was further categorized as participant-to-coach or coach-to-participant; phone calls with a coach were not recorded as an interaction. Program satisfaction was asked at week 12 (“On a scale of 0-10, how likely is it that you would recommend the Hinge Health program to a friend or colleague?”, 0-10).

### Statistical Analysis

The distribution of gender and BMI in the knee and back pathways were compared using 2-sided Fisher’s exact test and Mann-Whitney test, respectively. The association of baseline variables with program completion status was modeled using a logistic regression model and Wald’s confidence intervals for the odds ratios (ORs) estimated. Exploratory analyses visualized the relationship between overall pain reduction over the course of the DCP and the total number of ET sessions (grouped in equisized bins assuming an average of 35 ET sessions for program completers). VAS pain trends were modeled using piecewise linear regression splines. Intersubject variability in the rate of change was modeled through random effects and used a first-order autoregression correlation structure to model within-subject correlation in residuals. Optimal knot locations for the spline were determined by a cross-validation procedure that evaluated model fit on a grid of knot locations. The fixed effects were estimated using a linear mixed-effects model (Multimedia Appendix 3). Significance (*P* value) evaluation was based on Wald *t* values with a Satterthwaite correction. For pain-response subgroup analysis, a Gaussian mixture model was fitted to the estimated spline coefficients to discover clusters corresponding to subgroups within the cohort, each with a distinct pain reduction trend. Adjusted ORs were computed to understand the association between participants’ characteristics and the representative pain reduction trends for each subgroup. Finally, classification algorithms were trained to distinguish the 3 response groups based on the participants’ demographic and baseline measurements alone, and performance was evaluated using 5-fold cross-validation. All analyses were performed using R statistical computing software.

## Results

### Participant Demographics and Digital Care Program Completion

Of the 10,264 DCP participants, 6468 self-reported back pain and were enrolled in the back-pain pathway and 3796 self-reported knee pain and were enrolled in the knee-pain pathway. The average age was 43.6 years, and the average BMI was 30.25. The proportion of female participants in the DCP was 50.00% (5132/10,264). Compared with the back-pain pathway, BMI was 1.3 kg/m^2^ higher (*P*<.001) and the proportion of female participants was 3.9% higher (*P*<.001) in the knee-pain pathway. The difference in mean age between pathways was not significant (Table 1).

In all, 73.04% (7497/10,264) of the participants completed the DCP (referred to as *completers*), defined as completing at least one exercise session or reading 1 educational paper in weeks 9-12. Older users were more likely to complete the DCP (OR 1.037, 95% CI 1.03-1.04), whereas those with a higher BMI were less likely to complete the DCP (OR 0.973, 95% CI 0.97-0.98). No other baseline measures were significantly associated with completion (Multimedia Appendix 3). On average, completers engaged in 10.45 weeks with 35.02 ET sessions and 19.39 education sessions. Table 2 summarizes the engagement by pathway for all participants and completers. No injuries or other adverse effects of DCP engagement other than temporary discomfort were reported.

**Table 2:** Mean engagement and SD for the full cohort and for completers by pathway (N=10,264).

Variables	All			Completers		
	Overall	Back pain	Knee pain	Overall	Back pain	Knee pain
Number of participants, n (%)	10,264 (100.00)	6468 (63.02)	3796 (36.98)	7497 (73.04)	4676 (72.29)	2821 (74.32)
Weeks engaged (ET^a^ session or education session), mean (SD)	8.46 (3.9)	8.36 (3.92)	8.63 (3.86)	10.45 (2.15)	10.39 (2.17)	10.54 (2.1)
ET sessions per week, mean (SD)	2.93 (1.47)	2.85 (1.46)	3.05(1.47)	3.26 (1.39)	3.18 (1.41)	3.4 (1.34)
Total ET sessions, mean (SD)	27.43 (20.56)	26.48 (20.45)	29.04 (20.65)	35.02 (18.68)	34.04 (18.86)	36.65 (18.25)
Education sessions per week, mean (SD)	2.24 (1.55)	2.2 (1.55)	2.31 (1.56)	2.44 (1.28)	2.4 (1.27)	2.5 (1.3)
Total Education session, mean (SD)	15.33 (13.27)	14.81 (13.00)	16.24 (13.67)	19.39 (12.92)	18.84 (12.71)	20.29 (13.20)
Coach interactions per week, mean (SD)	7.03 (3.21)	6.99 (3.09)	7.09 (3.39)	7.23 (3.25)	7.21 (3.15)	7.27 (3.4)
Total coach interactions, mean (SD)	84.08 (43.3)	83.55 (42.02)	84.97 (45.36)	91.47 (43.42)	91.03 (42.33)	92.19 (45.16)

^a^ET: exercise therapy.

### Longitudinal Changes in Pain

**Figure 1:**
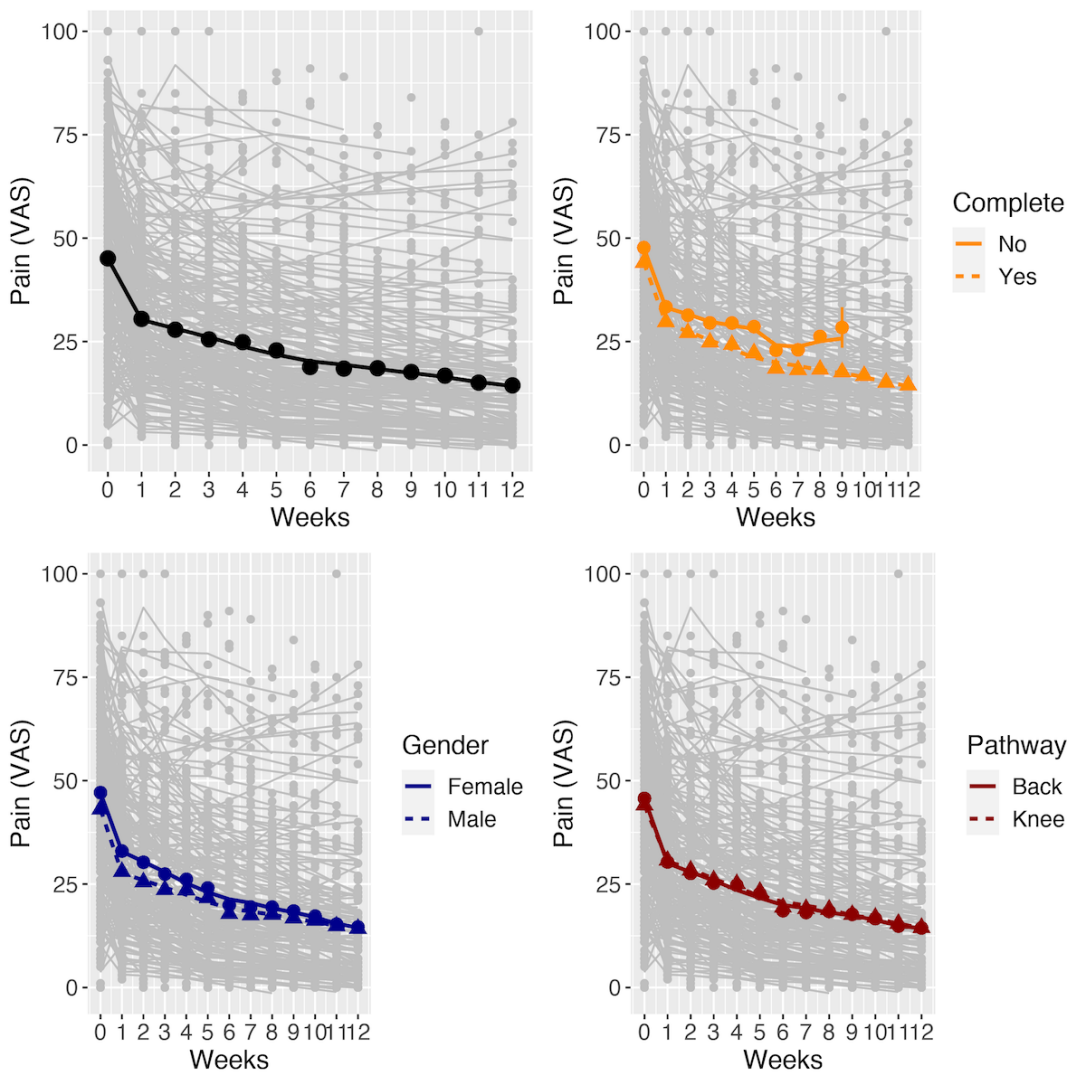
Longitudinal changes in pain. The panels show the average pain scores computed for the entire study cohort (circles) and the fitted means (lines) computed for weeks 0-12 of the study. Top left shows the overall fitted mean. The plots on the top right, bottom left and bottom right show the means for subjects grouped by completion status, gender, and pathway, respectively. Weekly recorded pain and fitted curves for a random sample of subjects are plotted in gray on each panel. Error bars indicate 1 SE of the mean. F: female; M: male; VAS: visual analog scale for pain.

On the basis of a linear mixed effects model, the estimated mean reduction in pain by week 12 was 68.45% (30.89 points). Participants’ pain scores changed nonlinearly over time (Figure 1). The mean change in pain scores per week (adjusted for sex, pathway, baseline age, BMI, anxiety, and depression scores) was 15.96 points for week 1 (*P*<.001) and 1.11 points per week for weeks 6-12 (*P*<.001) but was not significant for weeks 2-5. The conditional and marginal R-squared statistics [22] for our model were 0.94 and 0.54, respectively.

Minimally important change from baseline pain (defined as either a VAS pain reduction of 20 points or 30% with respect to baseline) was achieved by 78.60% (5893/7497) of completers and 69.60% (7144/10,264) of all participants.

Completers demonstrated greater pain reduction than noncompleters (Figure 1, top right) with an increased mean reduction rate of 0.48 points per week (SE 0.14) in weeks 2-5. Final pain reduction was nearly identical for both male and female genders (Figure 1, bottom left). However, there was a significantly higher mean reduction rate for male participants in the first week (mean difference=0.89 points per week, SE 0.46), and lower mean reduction rates in weeks 2-5 (difference=0.47 points per week, SE 0.09) and weeks 6-12 (difference=0.22 points per week, SE 0.05). Compared with the knee pathway, the back pathway was associated with a higher mean pain reduction rate (difference=3.1 points per week, SE 0.48) in the first week, but the pathway was not a significant variable in later weeks (Figure 1, bottom right).

### Effect From Engagement

**Figure 2:**
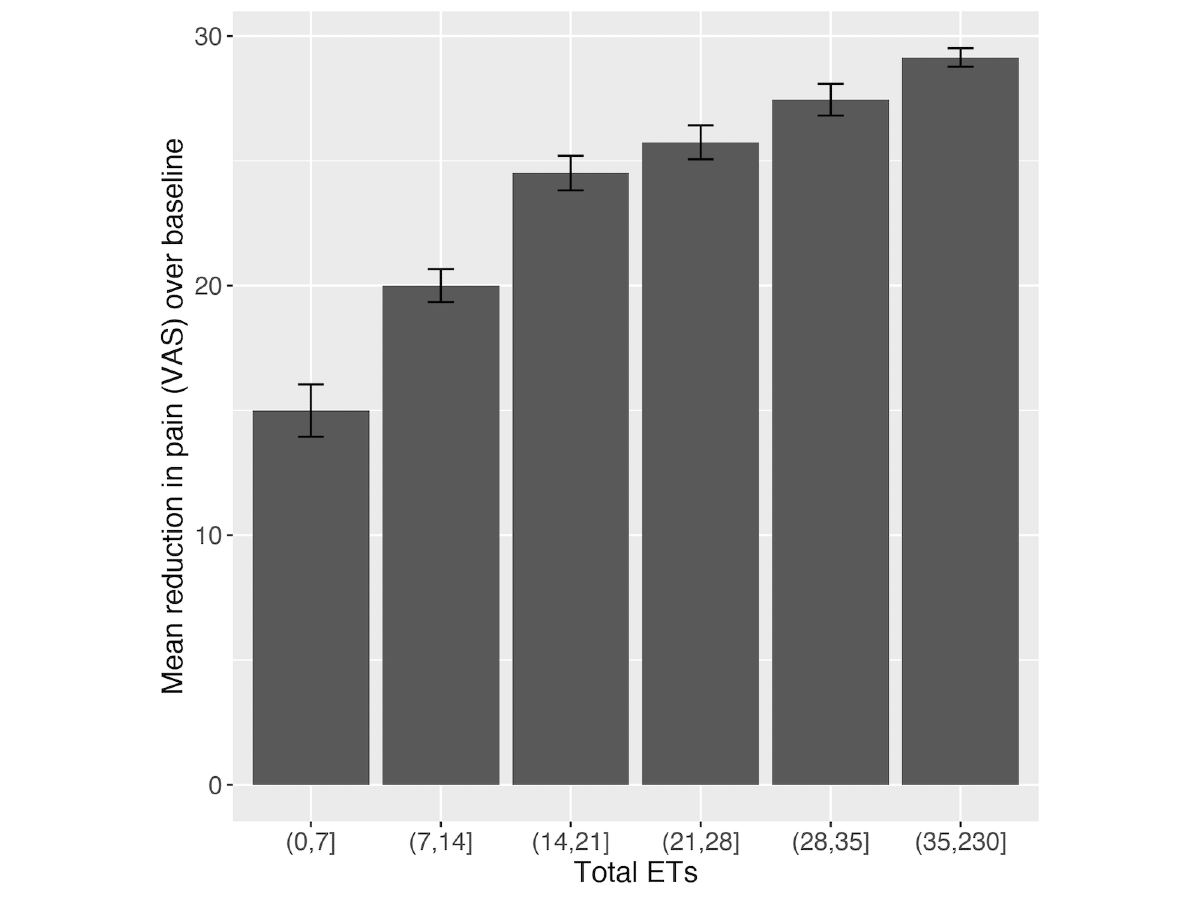
Association between pain reduction and ET sessions. Bar plots show the mean reduction in pain achieved over the DCP grouped by the total number of ET sessions. Error bars indicate 1 SE of the mean. DCP: digital care pathway; ET: exercise therapy; VAS: visual analog scale for pain.

Increasing levels of ET engagement in the DCP were associated with greater reductions in VAS pain score (*P*<.001; Figure 2). Notably, the relationship between the change in pain score and the number of ET sessions was nonlinear, with initial ET sessions contributing a higher proportion of the mean reduction achieved. The rate of reduction (adjusted for gender, pathway, baseline age, BMI, anxiety, and depression scores) for the initial 10 ET sessions was 1.9 VAS points per session (SE 0.2; *P*<.001).

The number of weekly coach interactions was also associated with a reduction in pain with a mean reduction of 0.18 VAS points per interaction (SE 0.06; *P*=.003) for the first 30 interactions. The number of participant-to-coach interactions, specifically, was associated with a mean rate of reduction in pain of 0.30 VAS points per interaction (SE 0.1; *P*=.003) for the first 20 interactions. The number of coach-to-participant interactions was not significantly associated with pain reduction.

### Mental Health and Other Secondary Outcome Measures

For participants categorized as having depressive symptoms (PHQ-9≥5) at baseline, the mean baseline PHQ-9 score was 12.01 and decreased by 57.9% to 5.05 at week 11 (*P*<.001). Differences between the pathways were not significant. The percentage of patients with depressive symptoms at baseline and at the end of the study was 21.1% and 11.4%, respectively. For participants categorized as having anxiety symptoms (GAD-7≥5) at baseline, the mean baseline GAD-7 score was 11.49 and decreased by 58.3% to 4.78 at week 11 (*P*<.001). The back pathway participants had a 0.46 point (*P*<.001) greater mean GAD-7 reduction than those in the knee pathway. The percentage of patients with anxiety symptoms at baseline and the end of the study was 28.3% and 14.2%, respectively (PHQ-9 and GAD-7 values at week 6 were carried forward to impute missing values at week 12).

With respect to baseline, the mean surgery likelihood score decreased by 67.4% (8.15 points, *P*<.001) overall, and by 66.8% and 68.2% for knee and back pathway participants, respectively. The mean KOOS—pain decreased by 33.9% (5.19 points, *P*<.001) in knee pathway participants and the mean MvK decreased by 51.4% (8.20 points, *P*<.001) in the back pathway participants. The within-participant correlation coefficients for KOOS—pain and MvK scores (with VAS pain) were 0.59 (95% CI 0.58-0.61) and 0.80 (95% CI 0.79, 0.81), respectively, indicating strong correlations between the primary and secondary pain variables. The mean WPAI score decreased by 63.94% from baseline (20.29 points, *P*<.001). The DCP final satisfaction score was 8.97/10 with a net promoter score of +64/100.

### Distinct Pain Response Groups

**Figure 3:**
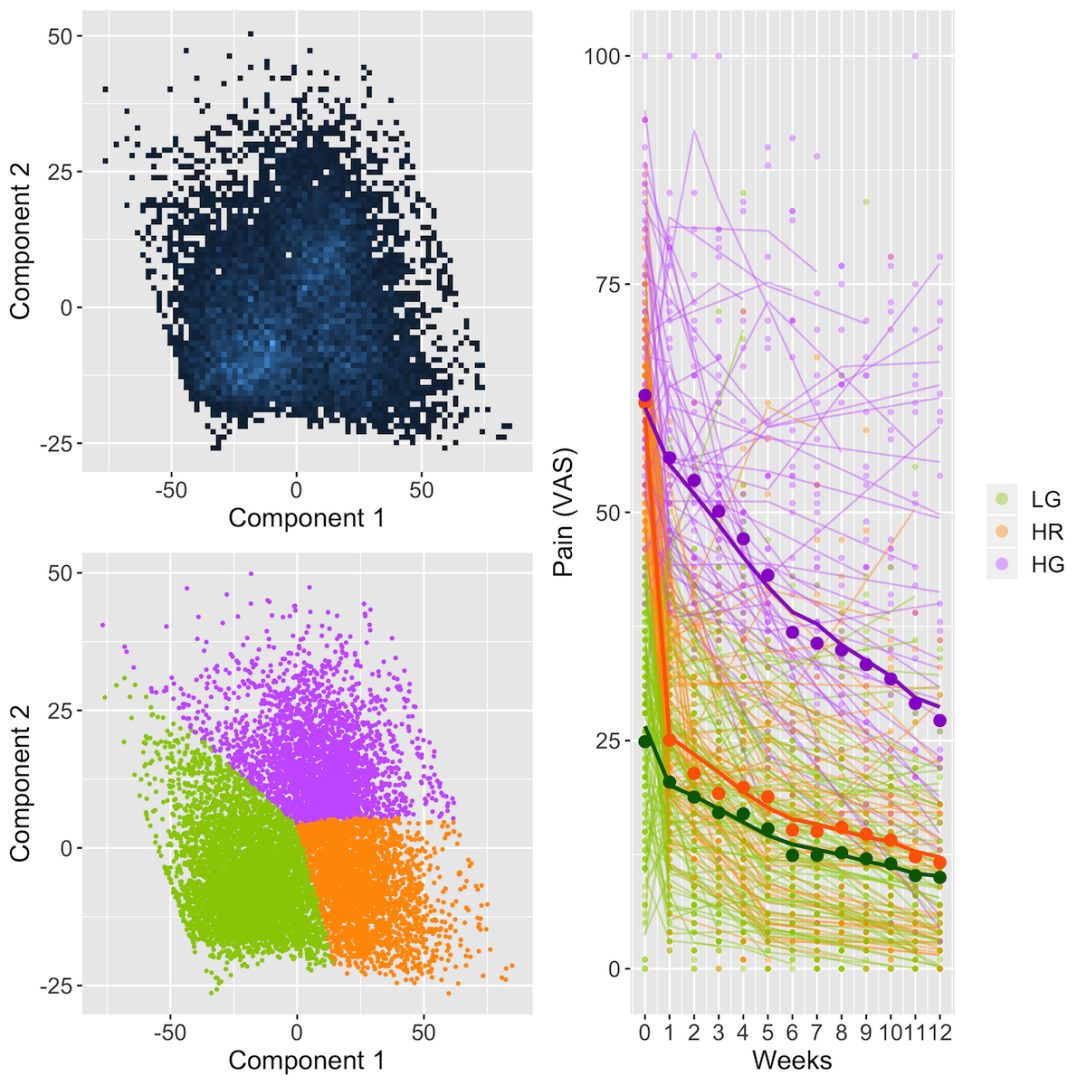
Pain response subgroups. Pain reduction trend clusters obtained by fitting a 3-component GMM identified 3 subgroups (HG, HR, and LG response). (Top left) 2D density plot of the first 2 principal components of the fitted splines shows each of the 3 subgroups. (Bottom left) Curves denoted by their respective principal components 1 and 2 are assigned to a cluster based on maximum posterior likelihood. (Right) Random sample of pain reduction trends colored by subgroup and the respective mean trends. 2D, 2 dimensional; GMM, Gaussian Mixture Model; HG, high-gradual; HR, high-rapid; LG: low-gradual.

Intersubject variation in pain reduction trends motivated a subgroup analysis of pain response, and 3 distinct response groups emerged (Figure 3). Participants with high pain at baseline and gradual improvement were designated as *high gradual* (HG). Participants with high baseline pain but a rapid decline were labeled *high rapid* (HR), and those with low baseline pain and gradual response were labeled *low gradual* (LG). All LG participants had baseline pain below 50. HR participants had the highest mean pain reduction over the duration of the DCP (48.8 points, 80.0%), followed by the HG (33.3 points, 54.1%) and LG group (15.3 points, 64.0%).

**Figure 4:**
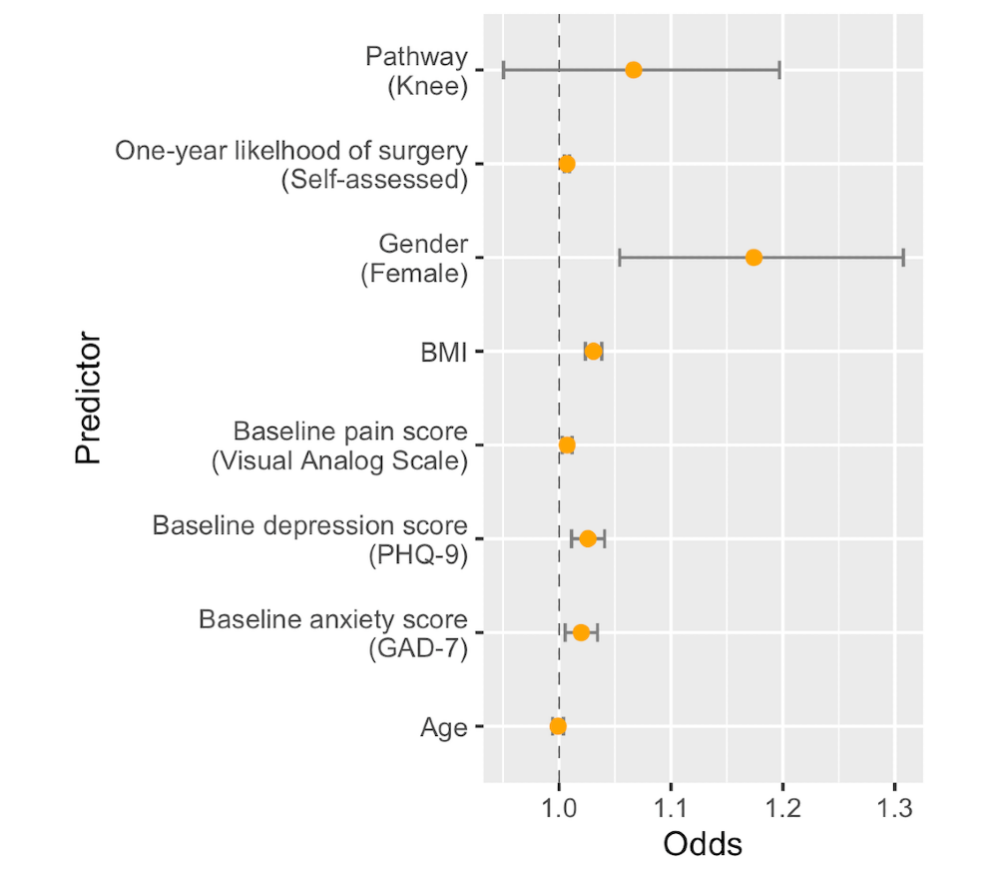
Association of baseline variables with a high-gradual or high-rapid pain reduction trend. For each baseline variable, the plotted values indicate the odds ratios for a gradual response (with reference to a high-rapid response) for a unit increase in the corresponding predictor. The error bars denote the 95% profile-likelihood CIs.

Relative to the HR response, female participants had 17.3% (*P*=.002) higher odds of an HG response (Figure 4). The odds of an HG response also increased by 3.1% (*P*<.001) per unit increase in BMI and increased by 2.2% (*P*=.001) and 2.1% (*P*=.002) per unit increase in PHQ-9 and GAD-7, respectively.

Classification of response groups based on baseline attributes achieved a mean accuracy of 76% (SE 0.3%) using a random forest algorithm, evaluated using 5-fold cross-validation. The classifier had a mean area under the precision-recall curve of 68.92% (SE 2.04%). Nearly equal numbers of participants belonged to the HR and HG response groups. Subgroup analysis details are provided in Multimedia Appendix 3.

## Discussion

### Principal Findings

This study demonstrated the positive effect of a 12-week DCP on chronic musculoskeletal pain outcomes in a large sample of real-world patients. Specifically, participants experienced a 68.5% average improvement in VAS pain between baseline and 12 weeks, and 78.60% (5893/7497) of program completers (7144/10,264, 69.60% of all participants) achieved clinically meaningful improvement. Completion was high, with 73.04% (7497/10,264) of participants reaching the final month, and completers engaged in a mean of 35.0 ET sessions, 19.4 education sessions, and 91.5 coach interactions during the DCP. It is well known that unless a digital health intervention fits into users’ daily lives, only a small proportion of all participants who sign up actually complete the program [23,24]. The exceptional completion rate of our study may be due to the multipronged strategy of our DCP that uses both a digital and a human interface to engage with participants. Furthermore, both the number of ET sessions and participant-to-coach interactions were positively associated with improvement in pain, supporting that the level of participant engagement influenced outcomes. These results support the effectiveness of a DCP for musculoskeletal pain in the real-world setting, and the large sample size supports the prospect for scalability to serve a large number of chronic low back and knee pain sufferers worldwide.

**Comparison With Literature**

The observed 68.5% average improvement in VAS pain in this DCP outperforms the pain reduction effect sizes observed in a variety of conservative care interventions with similar timeframes. For pain associated with knee osteoarthritis, comparable conservative care interventional studies demonstrate an average improvement in VAS pain of 19%-48% [25-28]. For low back pain, comparable studies demonstrate average improvements in VAS pain of 29%-53% [29-34]. Similarly, a systematic review of randomized clinical trials for low back pain showed a within-group standardized mean difference of 1.07 (95% CI 0.87-1.27) for pain reduction at 13 weeks [35], whereas a standardized mean difference of 1.37 (95% CI 1.33-1.40) for pain reduction at 12 weeks was observed in this study. Not only does this study demonstrate greater improvement in pain for both knee and low back pathways but it also has a much larger sample size than previous studies, which typically did not exceed 100 subjects. Furthermore, this study found a strong correlation between changes in VAS pain and secondary pain measures (KOOS—pain for knee and MvK for back), further supporting the validity of the VAS pain measurements. Finally, compared with other studies utilizing therapeutic exercise for chronic pain, this study demonstrated a similar lack of adverse events. This is likely attributable to the benefits and safety of light intensity stretching and strengthening exercises, and in this study may also be due to the exercise guidance provided by the wearable sensors.

### Patient Engagement

Notably, most previous studies have occurred in traditional clinical settings, where multiple barriers prevent both patients and clinicians from engaging in conservative care [36]. For example, adherence of chronic low back pain patients to home exercises prescribed from traditional physical therapy ranges from 30% to 50% and remains a significant challenge for administering effective care [37,38]. A primary benefit of a digital care approach for chronic musculoskeletal pain is the ability to engage patients with their treatment and self-management. Smartphone apps can cost-effectively deliver education and encourage healthy behaviors, whereas sensors can provide exercise guidance and track engagement [39]. The DCP in this study engaged 73.04% (7497/10,264) of users to completion, with completers engaging in 10.5 of the 12 weeks, including 3.3 ET sessions, 2.4 education papers, and 7.2 coach interactions per week (mean ET sessions 2.9, mean education sessions 2.2, and 7.0 coach interactions per week among all participants). Notably, this study demonstrated an association between pain improvement and both the number of ET sessions and the number of coach interactions, suggesting that the level of participant engagement impacted the results. Specifically, the first 10 ET sessions and the first 30 coach interactions were the most influential in pain improvement. Of note, a recent study evaluating a DCP in a similar population showed lower engagement and no relationship between exercise and pain reduction, suggesting that specific program implementation details (ie, sensor-guided exercises and health coaching) may have a large effect on outcomes [40].

### Mental Health Outcomes

Depression and anxiety are known to often occur in patients with chronic musculoskeletal pain [41], so the effects of this DCP on symptoms of depression and anxiety were also assessed. Behavioral health coaching and education on cognitive behavioral therapy concepts were key elements of the DCP’s multimodal digital care approach. A large body of research confirms the effect of psychological factors, such as depression and anxiety, on chronic pain [42,43]. In particular, an association between chronic low back pain and psychological factors has been shown, and related therapeutic approaches, including cognitive behavioral therapy and mindfulness-based stress reduction, have demonstrated effectiveness for back pain reduction [44]. This study showed that outcomes for participants with symptoms of depression and anxiety decreased on average by 57.9% and 58.3%, respectively, over the course of the DCP. This suggests a strong relationship between mental health and pain improvement; however, a causal relationship between these entities cannot be determined. Notably, mental health improvements were very similar across knee and back pathways, whereas a small difference (0.46 points) in GAD-7 outcomes was noted. This is unlikely to be clinically meaningful. Future work will further explore the effect of coaching and other behavioral health support on pain and functional outcomes.

### Predicting Pain Response

In addition to clinical effectiveness, another potential benefit of a DCP is the insight gained from longitudinal tracking of outcome data in large populations. The large sample size in this study, combined with data collection at regular and relatively frequent time intervals, enabled the discovery of distinct clusters of pain response trajectories over time. Participants were classified as gradual versus rapid pain responders, and patient-specific features that influenced the likelihood of pain response category were identified. By clustering distinct trends in pain response over time for each subject, we specifically uncovered 3 distinct pain response subgroups. Two groups had high baseline pain but differed in the rate of recovery (rapid vs gradual), whereas the third group had low baseline pain with gradual recovery. Notably, we were able to forecast with 76% accuracy which of these pain response groups a user would fall into based on their baseline information. Looking specifically at the 2 groups with high baseline pain, the rapid response was more likely to occur in male participants, those with lower BMI, or those with lower depression or anxiety scores. These pain response groups enable a better understanding of temporal changes in pain during the rehabilitation process and may ultimately help to identify pain recovery mechanisms. Furthermore, continued research into response patterns may ultimately allow for a more personalized approach to care, including more accurate prognosis and additional treatment options for patients likely to have a more gradual recovery.

### Strengths and Limitations

This study has several limitations, including the lack of a control group and the lack of physical function outcomes. Notably, previous randomized trials of this DCP on smaller populations (N<200) demonstrated positive effects on pain and functional outcomes (Oswestry disability index, KOOS—physical function short form) compared with control groups [17,18]. This study assessed outcomes in a sample of more than 10,000 users and demonstrated similar effectiveness. Another limitation of this study is the lack of long-term outcomes, and future studies should assess if participants are able to sustain healthy behaviors and self-management promoted in the DCP. Some potentially important demographic variables (ie, education, ethnicity, income, and smoking status) and medical history variables (ie, diabetes, hypertension, and mental health) were not obtained. Finally, this study was conducted through employers, which limits the applicability to clinical settings with higher proportions of uninsured, elderly, or work-disabled patients. However, this study was conducted with employees from 30 different companies across the United States and included a wide diversity of job types (eg, truck drivers, manual laborers, office workers), suggesting that the findings are applicable to a broad population. In addition, older patients were more likely to complete the program than younger ones, emphasizing that digital health tools are not only useful to the younger population.

The strengths of this study include the large sample size in the real-world setting, which demonstrated scalability and enabled the discovery of unique features, such as distinct pain response clusters in longitudinal real-world data. In addition, this study had similar age and sex distributions for knee and back pain participants, enabling direct comparison of the separate knee and back pathways. The average pain response for these separate pathways was quite similar (Figure 1), which is notable given the assumed underlying pathological differences between knee and back pain, but supports recent work urging practitioners to move beyond separating body regions when managing chronic musculoskeletal pain [45]. Finally, this study demonstrates significant improvements in self-reported workplace productivity (WPAI, 61.5% improvement) and surgery likelihood (67.4% reduction), suggesting that a DCP may have considerable economic benefits.

### Future Directions

DCPs may ultimately be used to complement clinical musculoskeletal practice, and further research is warranted on their use by patients and providers. This study supports the efficacy and scalability of a DCP for facilitating safe conservative care and promoting healthy behavior change. However, critical reviews have identified a lack of external and long-term validation of digital health tools [46]. Many previous studies on digital interventions for chronic low back pain have presented unconvincing results [47]. Given that digital health tools are typically developed in the private sector, and good clinical research can be time-consuming and challenging, we see a need for collaborative efforts between industry and academic medicine to optimize digital health technologies for effective conservative care implementation, adoption, and access in the broad, real-world population with musculoskeletal pain.

### Conclusions

This study supports the efficacy and scalability of a DCP for chronic low back and knee pain in a large, real-world population. Participants demonstrated very high completion and engagement rates, and a significant positive relationship between engagement and pain reduction was identified. This is the first longitudinal digital health study to analyze musculoskeletal health outcomes in a sample of this magnitude, and it supports the prospect for DCP scalability to serve the overwhelming number of chronic back and knee pain sufferers worldwide. Furthermore, the large sample size enabled the prediction of rapid versus gradual pain response from baseline information, which may prove beneficial for prognosis and tailoring future interventions. 

## Appendix

Multimedia Appendix 1 Hinge Health Digital Care Pathway.

Multimedia Appendix 2 Hinge Health Digital Back Pathway - woodpecker exercise.

Multimedia Appendix 3 Supplementary tables and figures.

## Abbreviations

DCP: digital care program
ET: exercise therapy
GAD-7: generalized anxiety disorder 7-item
HG: high gradual
HR: high rapid
KOOS—pain: knee injury and osteoarthritis outcome score—pain
LG: low gradual
MvK: Modified von Korff scale
OR: odds ratio
PHQ-9: patient health questionnaire 9-item scale
VAS: visual analog scale
WPAI: work productivity and activity impairment
