# Supplementary figures and images for: Digital Care for Chronic Musculoskeletal Pain: 10,000 Participant Longitudinal Cohort Study
Source: J Med Internet Res. 2020 May 11;22(5):e18250. doi: 10.2196/18250 (PMC7248800; doi:10.2196/18250)

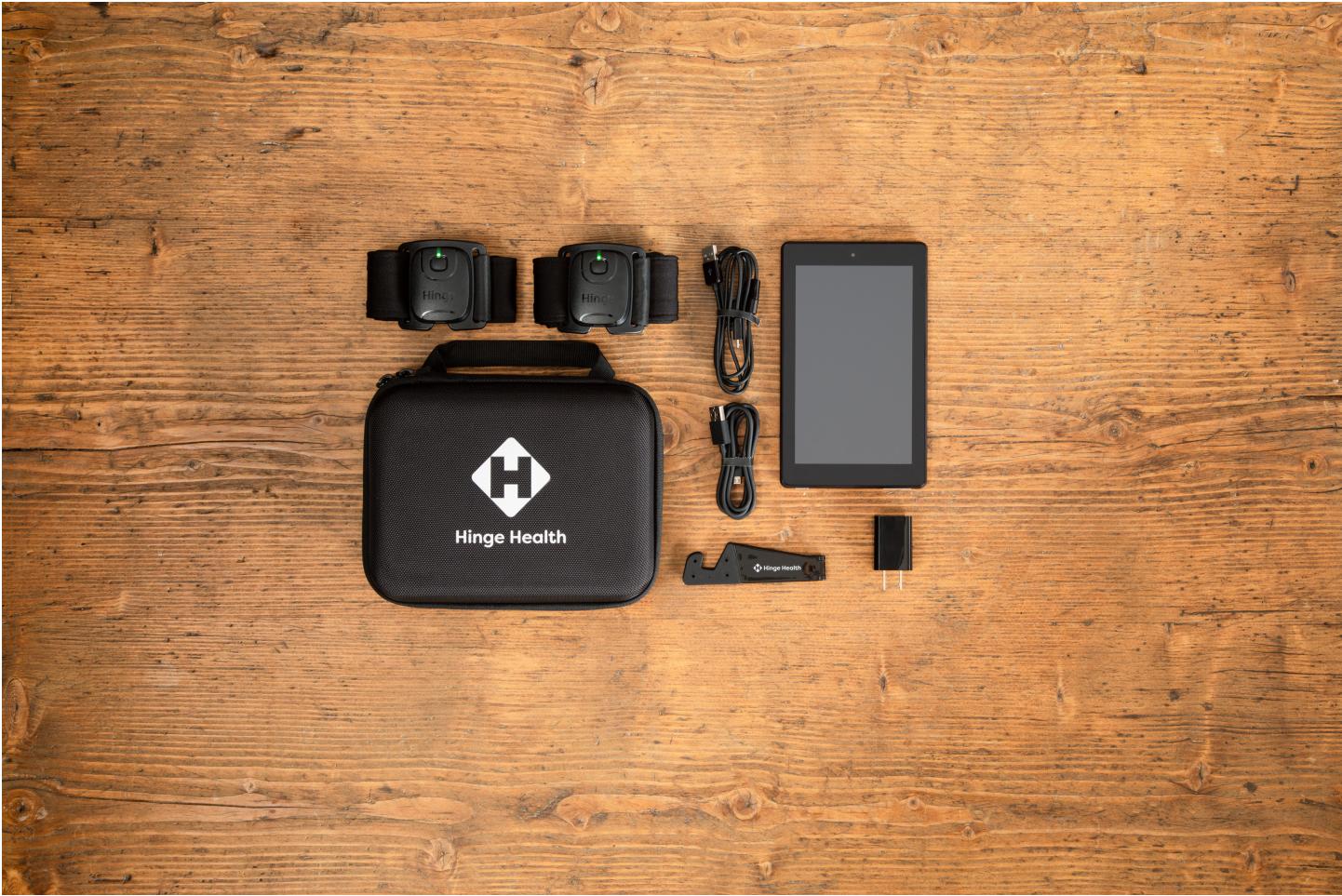

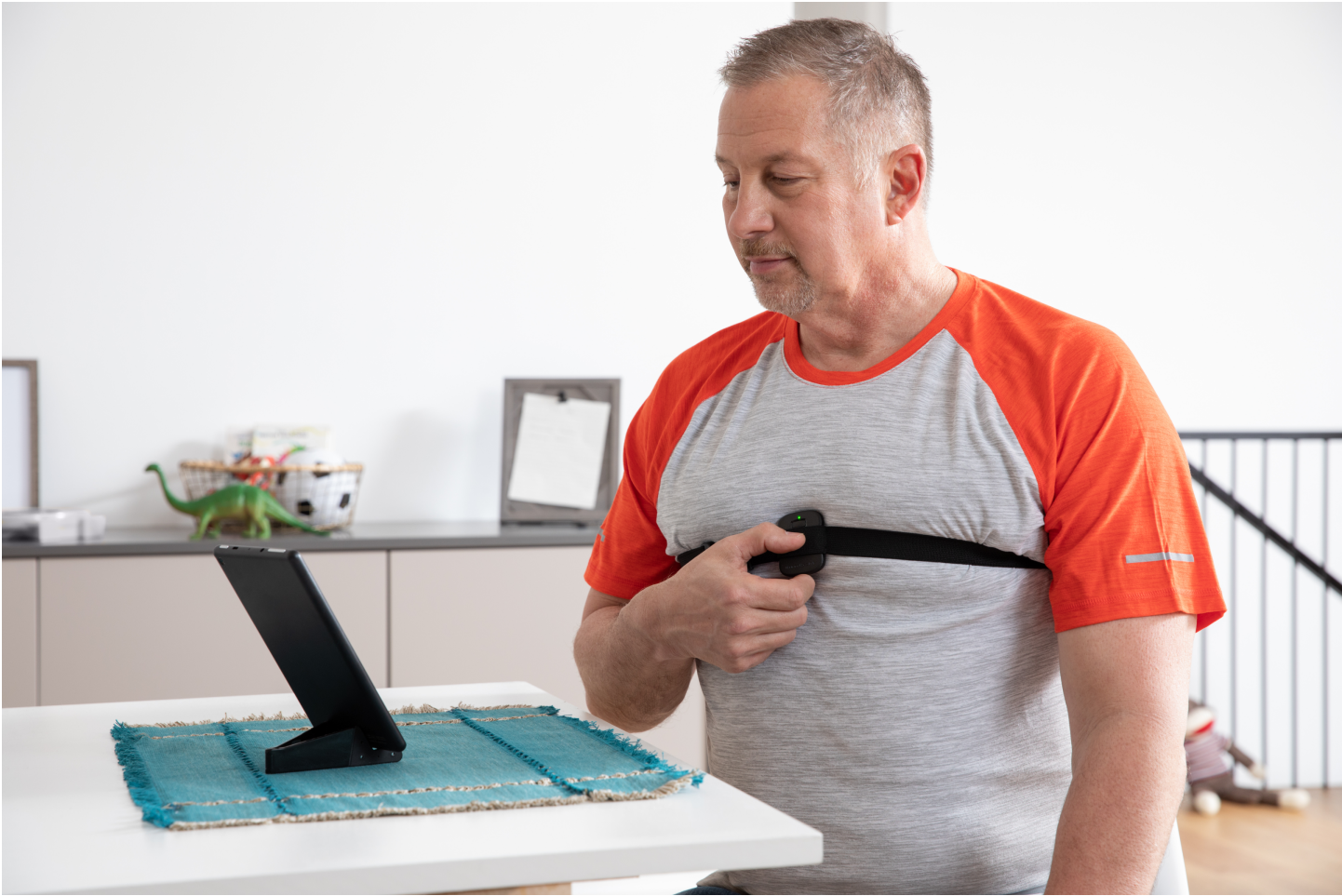

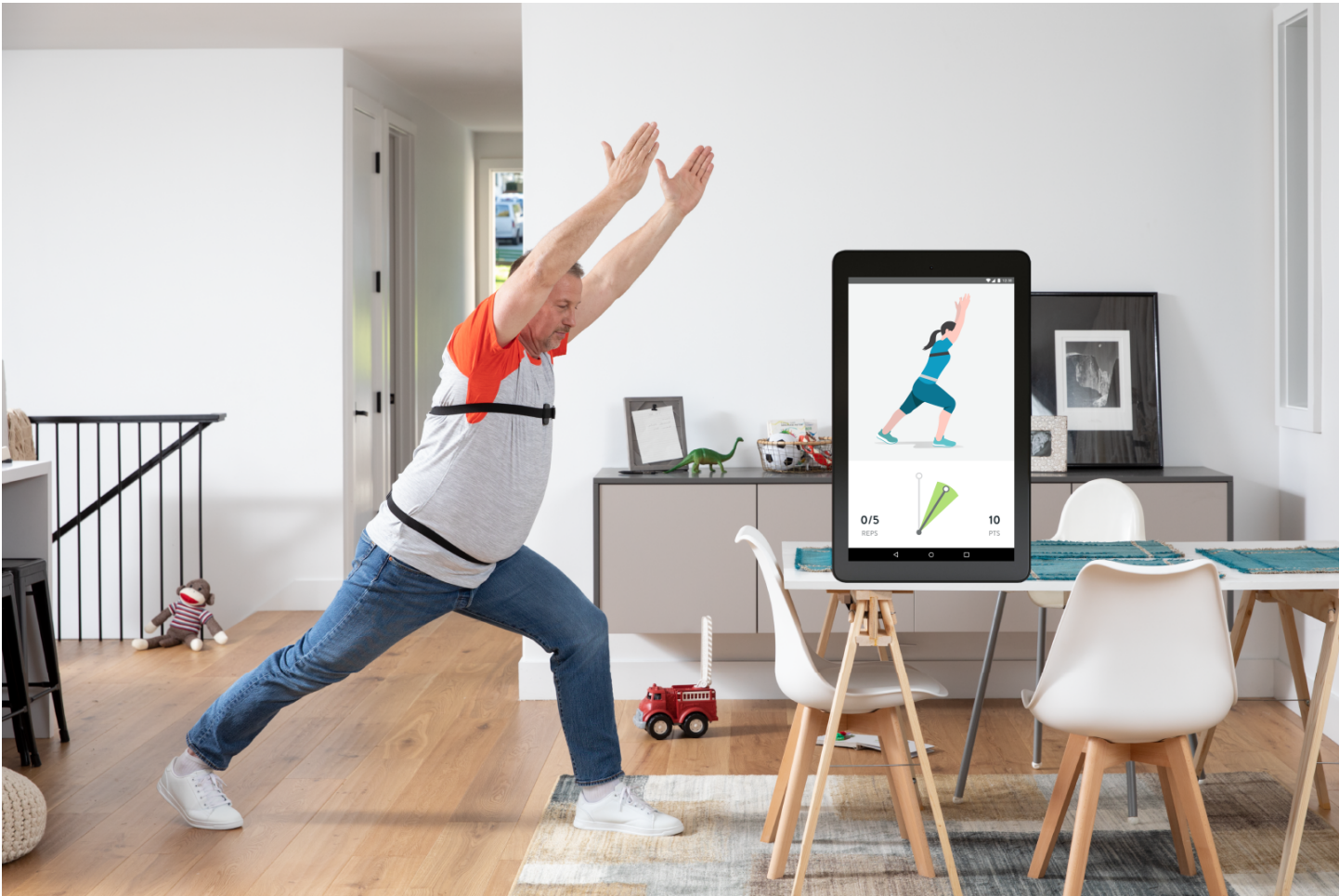

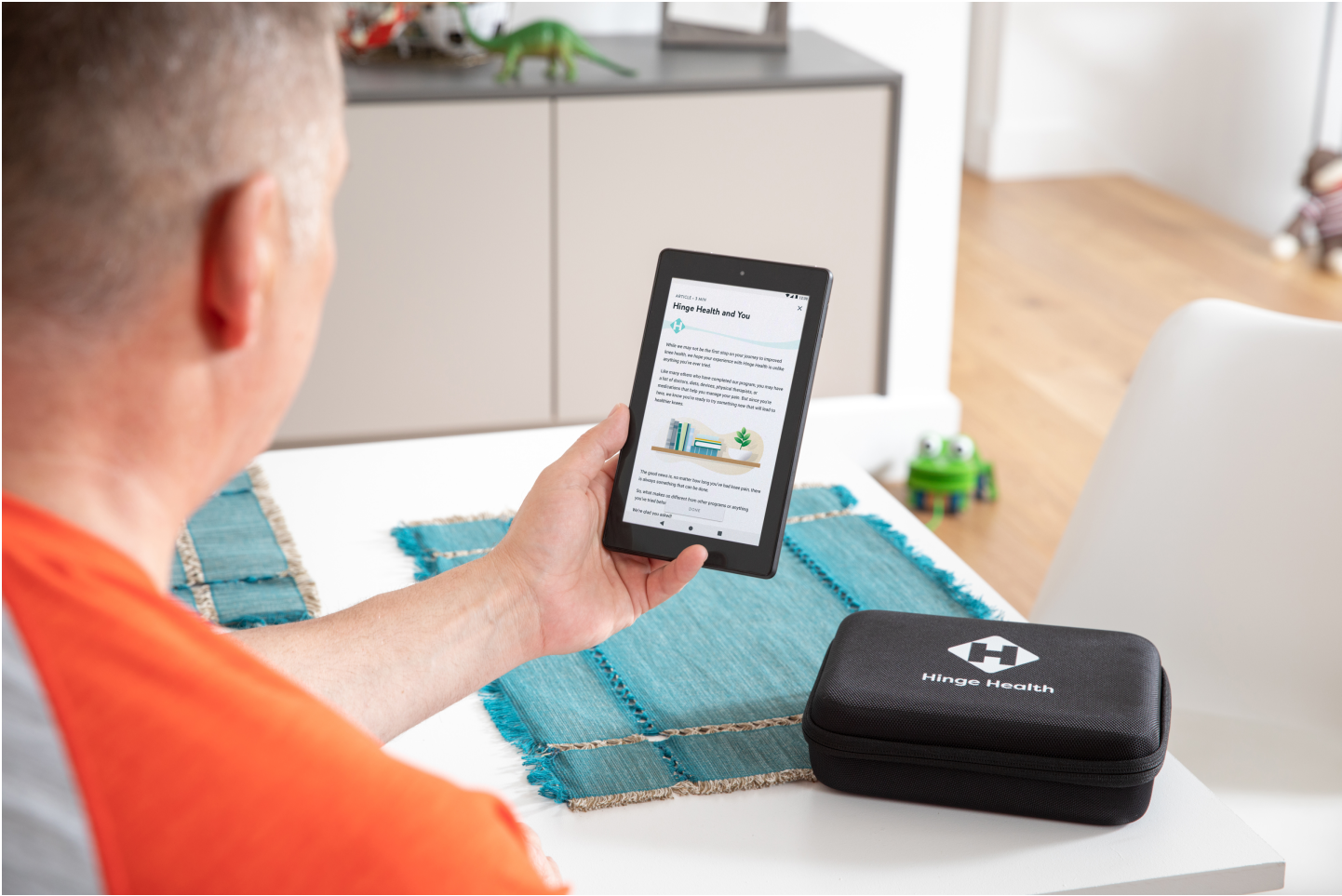

Supplement: Multimedia Appendix 1 [file jmir_v22i5e18250_app1.pdf]
