# Supplementary material for: Digital Care for Chronic Musculoskeletal Pain: 10,000 Participant Longitudinal Cohort Study
Source: J Med Internet Res. 2020 May 11;22(5):e18250. doi: 10.2196/18250 (PMC7248800; doi:10.2196/18250)

**Supplementary Information S1: DCP completion**

| Baseline variable | Log Odds of Completion | p-value |
| --- | --- | --- |
| age | 0.032 | < 0.0001 |
| pathway | 0.051 | 0.29 |
| gender | -0.078 | 0.09 |
| BMI | -0.022 | <0.0001 |
| VAS (baseline) | -0.006 | <0.0001 |
| Likelihood of surgery in 1 yr (baseline) | -0.004 | <0.001 |
| PHQ9 (baseline) | -0.008 | 0.21 |
| GAD7 (baseline) | -0.002 | 0.71 |

**Supplementary Information - S2**

Sub-group classification: Evaluation results based on 5-fold CV

|  | **Logistic Regression** | **K Nearest Neighbors (K=70)** | **Random Forest** |
| --- | --- | --- | --- |
| Classification acc (3 class) | 0.75 (0.004) | 0.75 (0.004) | 0.76 (0.003) |
| Classification acc (LG verus rest) | 0.96 (0.002) | 0.95 (0.002) | 0.96 (0.002) |
| Classification acc (HG verus rest) | 0.76 (0.004) | 0.76 (0.003) | 0.76 (0.002) |
| Classification acc (HR verus rest) | 0.79 (0.002) | 0.78 (0.004) | 0.79 (0.002) |
| Mean AUPRC | 0.61 (0.023) | - | 0.69 (0.02) |

Mean values by response group. * denotes a statistically significant difference (alpha = 0.05)

|  | **LG** | **HR** | **HG** |
| --- | --- | --- | --- |
| Age | 43.42 | 43.63 | 43.71 |
| BMI* | 29.28 | 29.99 | 32.27 |
| Gender (Female %)* | 45.47 | 48.52 | 59.49 |
| Baseline |  |  |  |
| Pain (VAS)* | 24.87 | 62.01 | 62.68 |
| PHQ-9* | 2.34 | 2.88 | 4.48 |
| GAD-7* | 3.28 | 3.75 | 5.24 |
| 1-year Surgery Likelihood*  (0 – 100) | 7.24 | 14.59 | 20.17 |
| Pathway (Knee %) | 39.17% | 33.43% | 37.03% |

**Supplementary Information – S3**

The linear mixed effects framework provides a way to estimate *individual* random effects (computed as the Best Linear Unbiased Predictors of random effects). Individual fits for a subset of participants are shown in


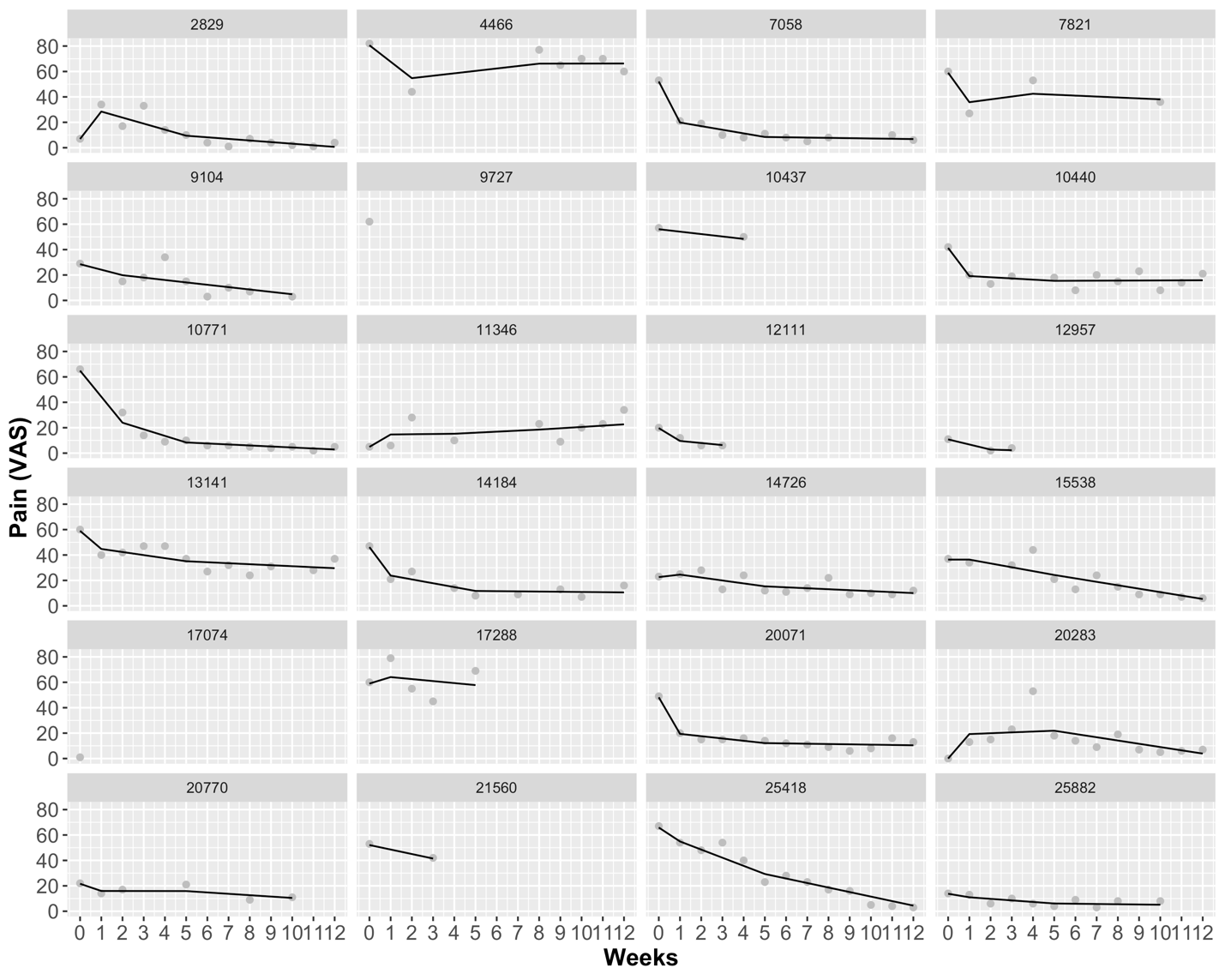

Supplement: Multimedia Appendix 3 [file jmir_v22i5e18250_app3.docx]
